# DNA immunoprecipitation semiconductor sequencing (DIP-SC-seq) as a rapid method to generate genome wide epigenetic signatures

**DOI:** 10.1038/srep09778

**Published:** 2015-05-14

**Authors:** John P. Thomson, Angie Fawkes, Raffaele Ottaviano, Jennifer M. Hunter, Ruchi Shukla, Heidi K. Mjoseng, Richard Clark, Audrey Coutts, Lee Murphy, Richard R. Meehan

**Affiliations:** 1 MRC Human Genetics Unit at the Institute of Genetics and Molecular Medicine at the University of Edinburgh, Crewe Road, Edinburgh, EH4 2XU, UK; 2 Wellcome Trust Clinical Research Facility, University of Edinburgh, Western General Hospital, Crewe Road, Edinburgh, UK

## Abstract

Modification of DNA resulting in 5-methylcytosine (5 mC) or 5-hydroxymethylcytosine (5hmC) has been shown to influence the local chromatin environment and affect transcription. Although recent advances in next generation sequencing technology allow researchers to map epigenetic modifications across the genome, such experiments are often time-consuming and cost prohibitive. Here we present a rapid and cost effective method of generating genome wide DNA modification maps utilising commercially available semiconductor based technology (DNA immunoprecipitation semiconductor sequencing; “DIP-SC-seq”) on the Ion Proton sequencer. Focussing on the 5hmC mark we demonstrate, by directly comparing with alternative sequencing strategies, that this platform can successfully generate genome wide 5hmC patterns from as little as 500 ng of genomic DNA in less than 4 days. Such a method can therefore facilitate the rapid generation of multiple genome wide epigenetic datasets.

The advent of next-generation sequencing (NGS) technology has resulted in a tremendous acceleration in the rates at which large sets of transcriptional, genetic and epigenetic data can be generated and analysed; impacting on a wide range of scientific disciplines^1^,^2^. Today’s sequencing based experiments typically generate more data than alternative methods such as microarray based assays; allowing the researcher to carry out high resolution analyses such as quantification of both transcriptional activity (RNA sequencing: RNAseq) and epigenetic modification patterns (DNA modification sequencing; “DIP-seq” and chromatin immunoprecipitation sequencing; “ChIP-seq”^3^,^4^,^5^. However the cost of generating replicates, compared to array based methods, can be steep. At present, Illumina based sequencing technologies (“Mi-seq” and “Hi-seq” DNA sequencers) are most frequently applied for the majority of genome wide studies. In 2010, Life Technologies developed a DNA sequencer which uses the principles of ion semiconductor sequencing^6^,^7^. In short, the DNA of interest is hybridised to a microwell plate (or “P1 chip”) prior to the addition of a specific deoxyribonucleotide triphosphate (dNTPs) in the presence of DNA polymerase. The reaction chamber is flooded with a single species of dNTPs. If the introduced dNTP is complimentary to the leading base on the template strand it is incorporated into a growing strand, which causes the release of a hydrogen ion, which triggers an ion-sensitive field-effect transistor (ISFET) sensor and the reaction continues (Supplemental Figure 1). Multiple dNTP molecules will be incorporated in a single cycle if runs of a particular dNTP are present leading to a corresponding number of released hydrogens and a proportionally higher electronic signal.

It was recently shown that “first generation” Ion Torrent sequencers were capable of accurately mapping the abundant histone H3 lysine 4 tri-methylation (H3K4me3) modification across the genome^8^. However this first generation technology is only able to sequence ~6 million reads in a single run – rendering it unable to reach the required read depth to sequence the far less discrete DNA modification patterns. With the recent development of a “second generation” semiconductor based sequencer (named the “Ion Proton sequencer”), which is capable of sequencing between 80–100 million reads per run, researchers are now able to explore the use of this relatively cost-effective and rapid sequencer for DNA modification based genome wide studies. In addition, semiconductor sequencing has the advantage of being able to sequence longer DNA fragments than alternative platforms (typically ~120 bp–150 bp (or greater) in length compared to Illumina 50 bp or 100 bp read lengths; Table 1) which increases the mapping efficiency greatly. Here we outline a method that utilises this commercially available semiconductor sequencing technology to generate genome wide patterns of DNA modifications following antibody immunoprecipitation; although this method will also work for 5hmC libraries generated by alternative enrichment strategies^4^,^9^. We focus in particular on profiling of the recently characterised and low abundant 5-hydroxymethylation (5hmC) mark, which constitutes between 0.1–7% of modified cytosine in non-CNS mouse tissues, the pattern of which is strongly perturbed in many cancers^10^,^11^,^12^,^13^,^14^. We have also successfully profiled the 5 mC mark in both tissue and cell lines using the same protocol (JT, unpublished data).

## Results

### Experimental design overview

Currently there are a host of methods to study the genome wide distributions of the 5hmC mark both at single base resolution^15^,^16^ and following enrichment for the 5hmC modification^4^,^13^,^17^,^18^,^19^,^20^. Due to the high cost of the former (a great degree of sequencing is required to generate sufficient coverage for such experiments), enrichment based assays (such as “affinity” enrichment or antibody based purification methods) are most routinely applied to study the distribution of 5hmC throughout the genome. Although these two techniques result in the generation of highly similar genome wide 5hmC patterns, a small number difference have been noted – particularly at CG poor loci and simple tandem repeats^9^,^21^. Due to the relative robustness of the protocol, high specificity of the antibody and overall quality of the resulting data^17^,^22^,^23^ we have adapted standard antibody enrichment protocols (hydroxymethyl DNA immunoproceipitation; hmeDIP) to generate genome wide datasets for 5hmC using a semiconductor sequencing approach which is both rapid, requiring under 4 hours to run and cost effective relative to alternative next generation sequencing strategies (table 1).

**Figure 1:**
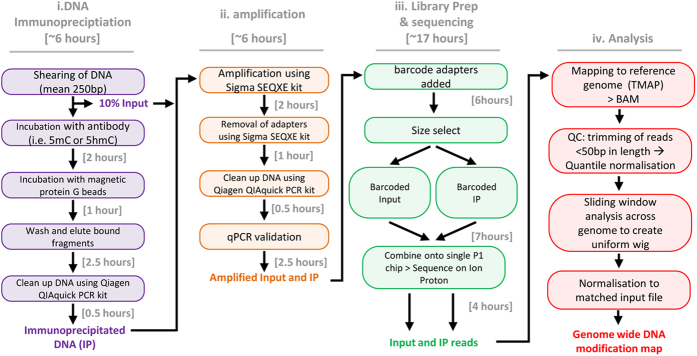
A flow-chart for the DNA immunoprecipitation semiconductor sequencing (DIP-SC-seq) protocol. Major steps in the DIP-SC-seq protocol are shown which can be broken into four stages. Step i is to enrich the DNA fragments of interest through antibody based pull downs. Step ii is low level whole genome amplification to generate double stranded DNA. Step iii is the preparation of sequencing libraries prior to sequencing on the semiconductor itself. And finally step iv is bioinformatic processing and normalisation. Overall it is possible to complete the entire protocol in less than 4 days.

The first phase of the hydroxymethyl DNA immunoprecipitation and semiconductor sequencing (“hmeDIP-SC-seq”) protocol relies on the fragmentation of high quality genomic DNA to generate a range of fragments 100–500 bp long, the majority of which are around 300 bp in length (Fig. 1). The next stage focusses on the enrichment of the fragmented DNA fragments which contain the epigenetic mark of interest (in this case 5hmC) using a highly specific antibody (for full protocol see^17^). It is also important that a reference input sample (10% of the total starting material) is removed prior to the enrichment steps and processed in parallel through the subsequent. This will allow the researcher to determine true enrichment over the background sequencing “noise”. In total these steps can take up to 6 hours to complete (Fig. 1).

**Figure 2:**
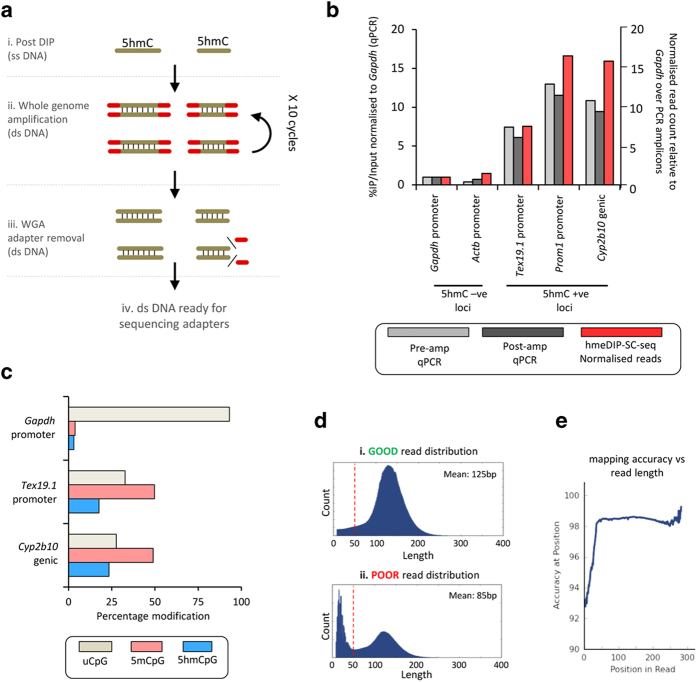
Whole genome amplification (WGA) as a method to generate double stranded DNA following DNA immunoprecipitation. (**a**) Schematic of the steps during the WGA. Red = WGA adapter sequences (**b**). Quantitative PCR (qPCR) analysis of several loci prior to WGA (post hmeDIP: light grey) as well as following WGA (dark grey) reveals little to no bias is introduced by the WGA. Analysis of the signals from the hmeDIP-SC-seq over these same regions highlights the retention of the patterns following sequencing (red bars). Loci were selected from previous work: 5hmC –ve = little/no 5hmC, 5hmC +ve = moderate/high 5hmC. Light grey bars: pre-amplified IP DNA, dark grey bars: post WGA DNA, red bars: normalised signals over locus used for qPCR taken from hmeDIP-SC-seq liver A dataset. qPCR data plotted against the primary y-axis, hmeDIP SC-seq data plotted against secondary y-axis (**c**). Glu-RES-qPCR results of three loci reveal quantitative information on 5hmC levels at a given site, confirming that pre and post WGA samples in fig. 2b reflect the expected 5hmC patterns. Tan: unmodified CpG, pink: methylated CpG, blue: hydroxymethylated CpG. (**d**) Examples of good and poor read length distributions following SC-seq. The majority of successfully sequenced DNA fragments should be >100 bp with a mean around 125–150 bp. (**e**) Plot of mapping efficiency vs read length. Reads less than 50 bp should be excluded due to poor mapping accuracy.

**Figure 3:**
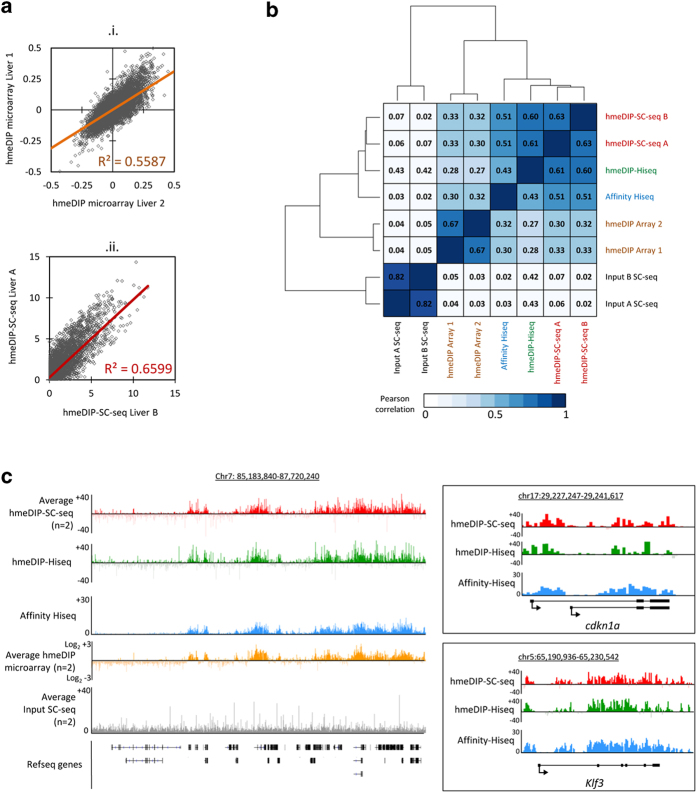
HmeDIP-SC-seq derived 5hmC data is comparable to published datasets using alternative profiling methodologies. (**a**) Scatter plots for 500,000 random 200 bp windows to plot biological replicate liver 5hmC levels for published hmeDIP tiled microarrays (i) or between hmeDIP-SC-seq (ii) datasets. R^2^ values are shown in each plot. For i, plots refer to log^2^ scores whilst ii refers to normalised average reads in a given 200 bp window. (**b**) Pearson’s correlation matrix with hierarchical clustering for replicate hmeDIP-SC-seq, input SC seq, hmeDIP-Hiseq, published affinity 5hmC Illumina Hiseq and 5hmC tiled microarray datasets. (**c**) Visual example of 5hmC patterns in the average input normalised hmeDIP-SC-seq (red), input normalised hmeDIP-Hiseq (green), published affinity Hiseq (blue) and published hmeDIP microarray datasets (gold) over a 2.5 Mb region on chromosome 7 (chr7:85,183,840–87,720,240). For a comparison, average input SC-seq data is shown in grey below. On the right, comparative 5hmC profiles are shown for hmeDIP-SC-seq and 5hmC affinity Hi-seq datasets over select loci (*Cdkn1a* and *Klf3*).

One notable downside of using the hmeDIP protocol is that the DNA must be denatured in order to facilitate efficient binding of the antibody. This results in an enriched library of single stranded DNA fragments. However in order to add specific adapter sequences to the ends of the DNA, the DNA must be double stranded. For this reason sequencing adaptors have been traditionally added to the genomic DNA prior to the enrichment steps, which is an expensive and somewhat technically challenging process. In the protocols outlined here we do not add the adapter sequences until after the enrichment stages. This is achieved by converting the single stranded DNA fragments enriched by hmeDIP into double stranded fragments by carrying out a low number of whole genome amplification (WGA) cycles using a commercially available method optimised for next generational sequencing (Sigma Aldrich enhanced amplification kit) (Fig. 2a). As this amplification requires the addition of its own adapter sequences, these are specifically cleaved off following amplification in order to increase the read quality from subsequent sequencing runs. Through comparison of our pre- and post- whole genome amplified material by qPCR over positive and negative control regions for 5hmC candidate loci (fig. 3b), which we independently validate using a glucosyl-sensitive restriction enzyme digestion assay (gRES-qPCR: see supplementary information) we do not find a significant degree of amplification bias introduced through such steps (Fig. 2b,c).

Following validation by quantitative PCR (qPCR) over known positive and negative control loci (Fig. 2b), the next stage is to prepare libraries from the input and enriched (or immunoprecipitated; “IP”) samples by the addition of specific barcoded adapter sequences (see online methods; ~6 hours), prior to template preparation (~7 hours) and sequencing on Ion P1 chips (~4 hours, (see online methods for more information)). Finally the reads are then mapped to the reference genome and basic bioinformatic normalisation and processing applied to generate genome wide patterns of the DNA modification of interest. If desired, it is possible to sequence a matched input for each IP on a single P1 chip (using specific barcoded sequencing adapters) allowing for an internal normalisation much like microarray technology does^24^. In addition to revealing enrichment values over the background sequencing noise, such normalisation will allow the direct comparison of datasets from karyotypically different sources. The resulting datasets can therefore either represent total reads across the genome (IP only) or total reads relative to input across the genome (a ratio of the IP reads vs the Input reads).

### Considerations to note following semiconductor sequencing

The amount of coverage (or depth) required for a sequencing experiment will be critical for the overall cost of the project. Traditionally this value refers to the number of times a nucleotide is read, versus the entire genome length. However for epigenetic based assays such as for 5hmC, true coverage values can be hard to achieve due to the fact that only select sites (certain CpG dinucleotides) are marked by the modification. For a single hmeDIP-SC-seq experiment (i.e. hmeDIP and matched input samples both sequenced on a single P1 microwell), between 70 and 80 million high quality reads are typically achieved (~35–40 million per IP or input sample. Table 1). We find that this number of reads, combined with the fact that the read lengths are frequently 120–150 bp long, results in comparable coverage values to similar published 5hmC and 5 mC DIP Illumina sequenced datasets^25^ (Supplementary Table 1).

Not only does the long read length increase the potential depth of the experiment but also results in high mapping efficiencies (typically >90% of reads mapped to the reference genome). To achieve optimal sequencing it is therefore important to check the size range of the sequenced DNA fragments. This will depend heavily on i) how well the initial gDNA was sheared prior to the DIP, ii) on the size selection imposed following the addition of the specific sequencing adapters and iii) on the quality of the DNA sequence (Fig. 2d). If the DNA sequence is of poor quality or contains left over WGA adapter sequence at its ends, the sequence will fall below the required QC cut-offs and the bases will not be included. As such these poor quality or repetitive bases will be removed resulting in smaller recorded sequences than expected (typically less than 50 bp in length). Additionally, degraded DNA fragments or residual unincorporated WGA adapter sequences will also be found in the pool of sequences less than 50 bp if these are not properly removed in the size selection step. Sequences less than 50 bp in length exhibit lower mapping accuracy scores than the longer fragments (Fig. 2e).

### Proof of principle: analysis of semiconductor derived hmeDIP-seq genome wide mouse liver 5hmC patterns

To test the utility of the Ion Proton as a method of generating genome wide DNA modification patterns we chose to characterise the low abundant 5hmC modification in mouse liver DNA; a modification and a tissue which are suitable for this type of analysis^9^,^23^,^26^,^27^,^28^. We directly compared the genome wide sequencing patterns for mouse liver hmeDIP libraries sequenced either on a single P1 Ion proton microwell or Illumina Hiseq 2000 lane (50 bp single end reads) as well as comparing to a published mouse liver 5hmC affinity enriched Hiseq dataset^29^ in which 5hmC marked DNA is first glucosylated and then captured using modified biotin beads^4^. In addition, we also carried out comparative analysis against tiled microarray datasets which were generated through hmeDIP enrichment^9^. To test for reproducibility of the semiconductor sequencing we carried out biological replicate runs on two mouse livers. To allow for direct comparisons of the datasets and to smooth the data we carried out sliding window analysis (*see methods*) with a window length of 200 bp.

Comparisons of the overall correlations between the probe values generated on biological replicate mouse livers on the tiled microarrays as well as the semiconductor reveals that both are similarly reproducible (R^2^ values of 0.5587 for the hmeDIP microarrays and 0.6599 for the hmeDIP-SC-seq runs with Pearson’s correlations of 0.6712 and 0.6327 respectively; Fig. 3a,b). Analysis of the overall Pearson’s correlations between all of the datasets reveals that genome wide liver 5hmC profiles by hmeDIP-SC-seq are similar to those sequenced on the Hiseq – both following hmeDIP and affinity enrichment strategies (Fig. 3b). In fact the hmeDIP enriched datasets clustered closer together than the chemical affinity method, arguing that enrichment method supersedes sequencing strategy as a means of generating comparable 5hmC patterns. As expected the microarrays cluster separately from the genome wide sequencing datasets. Although the two input (genomic sheared liver DNA) lanes were highly correlated with each other (Pearson’s correlation of 0.82) they displayed no correlation to the hmeDIP-SC-seq datasets (Pearson’s correlations < 0.07). Such a result reveals that the 5hmC patterns generated by the hmeDIP-SC-seq protocol are not strongly influenced by an inherent background sequencing bias. Visualisation of the datasets reinforces the use of the Ion torrent Proton technology as a suitable sequencer to map epigenetic marks; with similar patterns observed between the average hmeDIP-SC-seq, hmeDIP-Hiseq, Affinity Hiseq and average hmeDIP microarrays are shown in Fig. 3c.

**Figure 4:**
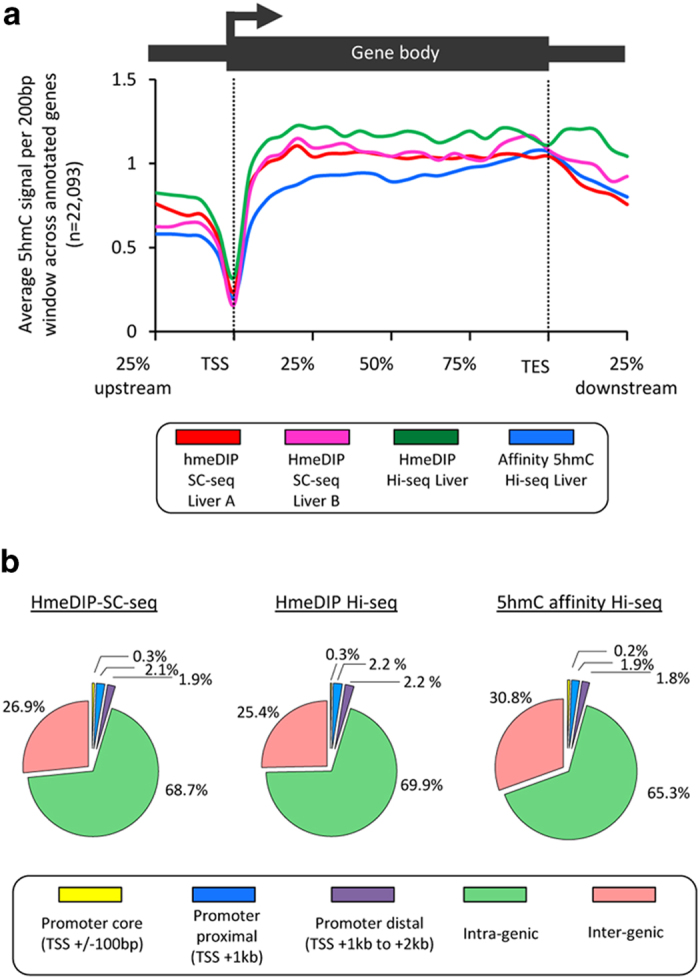
HmeDIP-SC-seq derived 5hmC patterns closely resemble those derived through sequencing on the Illumina Hiseq platform. (**a**) The average 5hmC patterns across genic portions of the genome are plotted for replicate hmeDIP-SC-seq runs (red and pink) alongside those for hmeDIP-Hiseq (green) and affinity-Hiseq (blue). The average number of reads in a 200 bp window are then calculated across each gene before averaging across 22,093 genes loci. Genic loci are extended 25% upstream and downstream of each region to analyse the surrounding environment. (**b**) The genomic distributions of 5hmC enriched windows (“peaks”. see supplementary information) in the hmeDIP-SC-seq, hmeDIP-Hiseq and published affinity Hi-seq datasets were compared by mapping to one of the five indicated regions in the genome. Percentage of total peaks mapping to each locus are shown.

It has been widely reported that the majority of 5hmC resides in the bodies of genes and promoter proximal regions of the genome whilst being depleted directly over the transcriptional start sites (TSS; for a review see^13^). Focusing on genic regions, we find that the 5hmC patterns generated though hmeDIP-SC-seq are in agreement with these findings and to patterns generated by hmeDIP-Hiseq and affinity Hiseq (Fig. 4a). Although there is a slight difference in the levels of 5hmC at the start of the gene body these differences appear to be related to the enrichment technique employed as they are specific to the affinity enriched 5hmC dataset. We then compare global distributions of the 5hmC modification through hmeDIP-SC-seq, hmeDIP-Hiseq and affinity-Hiseq routes by calling “peaks” of enrichment in the sequencing datasets (see online methods) which results in a binary output of the most strongly 5hmC modified regions. Mapping of these peaks to one of 5 compartments of the genome (promoter core, promoter proximal, promoter distal, intra-genic and inter-genic; see online methods) reveal a very similar genomic distribution of 5hmC marked regions in all three datasets (Fig. 4b).

**Figure 5:**
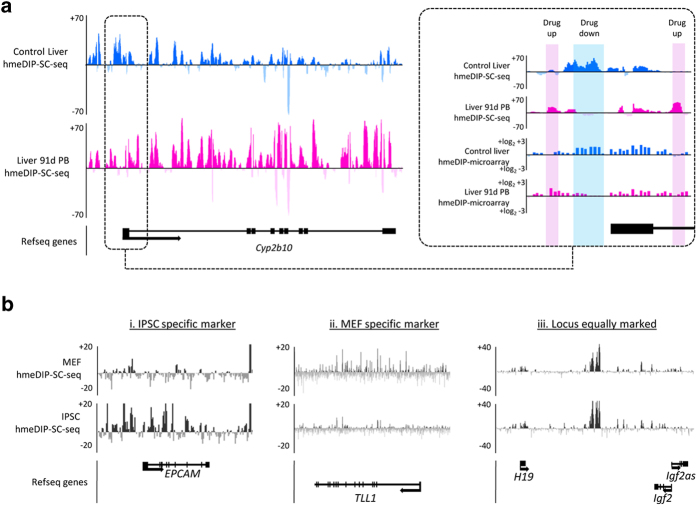
Examples of the utility of DIP-SC-seq. (**a**) 5hmC patterns generated by hmeDIP-SC-seq reflect toxicological insult over the *Cyp2b10* locus. Genic 5hmC patterns are elevated following 91 days phenobarbital (PB) exposure (pink) compared to the control liver 5hmC (blue). This reflects a strong elevation in gene expression from this locus (data not shown). Box on the left highlights the promoter region of the *Cyp2b10* gene and compares hmeDIP-SC-seq patterns to published 5hmC promoter microarrays. Regions which gain 5hmC upon drug expose are highlighted pink, regions which lose 5hmC are highlighted blue. (**b**) 5hmC patterns generated by hmeDIP-SC-seq can be used to track epigenetic changes which occur during reprogramming in cultured cells. Analysis of the 5hmC patterns over several candidate loci reveal IPSC specific loci (i), MEF specific loci (ii) as well as those which share a common 5hmC pattern (iii).

In addition to comparing to alternative sequencing strategies, we demonstrate the utility of hmeDIP-SC-seq as a tool for epigenetic analysis through two independent systems (Fig. 5). Firstly through the analysis of epigenetic toxicological insult in the mouse liver following long term drug exposure, and secondly in cell culture assays and the analysis of epigenetic differences which arise during reprogramming to induced pluripotent stem cells (IPSCs). Using promoter specific microarrays we have previously shown that exposure to the non-genotoxic carcinogen, phenobarbital (PB), results in an altered 5hmC landscape in the mouse liver^23^,^27^,^28^. In particular, the gene *Cyp2b10*, is both strongly induced to express and undergoes epigenetic change following PB exposure. Through hmeDIP-SC-seq we are able to validate these promoter specific changes as well as report on a strong genic elevation of 5hmC in the drug exposed animal; revealing a novel use for semiconductor sequencing in toxicity testing (Fig. 5a). Finally it was recently shown that 5hmC patterns may be used to identify cells which have been successfully reprogrammed into IPCs^30^. With this in mind we carried out hmeDIP-SC-seq on mouse IPSCs as well as the embryonic fibroblasts (MEFs) from which they were derived. Analysis of the resulting 5hmC patterns over select loci identified as being expressed exclusively in the MEFs or IPSCs^31^ reveals that such loci also have strong differences in their 5hmC patterns; once more highlighting the utility of hmeDIP-SC-seq as an analytical tool (Fig. 5b).

## Discussion

An overwhelming degree of evidence, most recently fuelled by advances in NGS technology, suggests that changes to epigenetic patterns can reflect the underlying gene expression patterns of a given cell and as such are fundamental to the overall cellular identity of the cell (for a review see)^32^,^33^. In addition, the epigenome can become perturbed in response to environmental change and toxicological insult^23^ as well as altered in a range of developmental disorders and diseases such as in cancer progression^34^. The advent of next generation sequencing technology has allowed researchers to study these epigenomes in more detail and attempt to better understand the epigenetic and molecular events which dynamically change during disease progression. However this approach has several drawbacks; largely the high costs associated with library preparation and subsequent sequencing, as well as relatively lengthy sequencing times. The high start-up costs of purchasing such hardware means that many researchers instead turn to biotech partners to sequence their DNA rather than attempt to do it in house, further adding to processing times. With this in mind we have optimised a simple protocol to utilise the Ion Torrent Proton semiconductor sequencer (Life tech) to generate genome wide profiles of epigenetic modifications. Focussing on the 5hmC DNA modification we have developed a protocol based on antibody enrichment followed by semiconductor sequencing (hydroxymethyl DNA immunoprecipitation semiconductor sequencing; “hmeDIP-SC-seq”) which returns reproducible patterns of the 5hmC mark that are comparable to datasets derived through Illumina based sequencing methods as well as high resolution microarray approaches. This case study investigating 5hmC profiles in liver DNA reveals the utility of the Ion Proton sequencer as an accurate, reproducible, rapid and cost effective strategy for DIP-seq and ChIP-seq projects.

## Methods

Full methods and any associated references are available in supplementary information.

### Overview of hmeDIP-SC-seq

>500 ng of genomic DNA is sheared to ~300 bp long fragments prior to immunoprecipitation (IP) with antibodies against 5hmC (Active motif rabbit polyclonal against hydroxymethylation cat#39769). For full protocols see^17^. 10% of the input is also taken prior to immunoprecipitation. In all stages DNA was purified using DNA Clean & Concentrator™ (Zymo Research).The IP and input samples undergo 10 cycles of whole genome amplification following associated protocols (Sigma-Aldrich SeqPlex DNA Amplification Kit). Sequencing libraries are made from 100 ng of DNA for each sample, using the Ion XpressPlus Fragment Library Kit (life Technologies™). The DNA was end repaired, purified and then ligated to Ion-compatible barcoded adapters (Ion Xpress™ Barcode Adapters 1–96: Life Technologies™), followed by nick-repair to complete the linkage between adapters and DNA inserts. The adapter-ligated library was then amplified (10 cycles) and finally size-selected using two rounds of AMPure XP bead capture to size‐select fragments approximately 100–250 bp in length. Samples were then pooled in a 1:1 ratio and sequenced on a single Ion Proton P1 microwell chip (Life Technologies™). Sequence reads were mapped to the reference genome using the supplied TMAP mapping software and the resulting BAM file processed for downstream data analysis.

## Author Contributions

Conceived and designed the experiments: J.T. and R.M. Performed the experiments: J.T., R.O., A.F., R.C., R.S., H.M., J.H. & A.C. Analysed the data: J.T. Wrote the article: J.T., L.M. & R.M.

## Accession codes

HmeDIP-SC-seq data can be found at the Gene accession omnibus (GEO) under the accession number GSE66889. High resolution tiled microarray datasets can be found on GEO under accession numbers GSE51577 and GSE40540. Affinity enriched 5hmC Illumina Hiseq datasets can be found on GEO under accession number GSE44566

## Additional Information

**How to cite this article**: Thomson, J. P. *et al.* DNA immunoprecipitation semiconductor sequencing (DIP-SC-seq) as a rapid method to generate genome wide epigenetic signatures. *Sci. Rep.* **5**, 9778; doi: 10.1038/srep09778 (2015).

## Supplementary Material

Supplementary Information

## Figures and Tables

**Table 1:** 

	**Ion Proton P1**	**Illumina Hiseq 2000**	
start up sequencer costs	~$250,000 (USD)	~ $650,000 (USD)	
ave read length	125bp	35bp (single end)	100bp x2 (paired end)
sequencing adapter cost	LOW (added post enrichment to ng DNA)	HIGHER (added pre enrichment to μg of DNA)	HIGHER (added pre enrichment to μg of DNA)
library prep and sequencing cost	comparable	comparable	comparable
total GB data per run	6-10Gb	26-35Gb	200Gb
time to sequence	4 hrs	36 hrs	192 hrs
maximum GB per hour	2.5 Gb/hr	0.97 Gb/hr	1.04 Gb/hr
Time to 100 Gb	40 hrs	103 hrs	96.1 hrs
