# Supplementary material for: DNA immunoprecipitation semiconductor sequencing (DIP-SC-seq) as a rapid method to generate genome wide epigenetic signatures
Source: Sci Rep. 2015 May 14;5:9778. doi: 10.1038/srep09778 (PMC4435000; doi:10.1038/srep09778)
Supplement: Supplementary Information [file srep09778-s1.pdf]

Title: DNA immunoprecipitation semiconductor sequencing (DIP-SC-seq) as a rapid method to generate genome wide epigenetic signatures

Authors: John P Thomson[1], Angie Fawkes[2], Raffaele Ottaviano[1], Jennifer M Hunter[1], Ruchi Shukla[1], Heidi K. Mjoseng[1], Richard Clark[2], Audrey Coutts[2], Lee Murphy[2] & Richard R Meehan[1]

1. MRC Human Genetics Unit at the Institute of Genetics and Molecular Medicine at the University of Edinburgh, Crewe Road, Edinburgh, EH4 2XU, UK

2. Wellcome Trust Clinical Research Facility, University of Edinburgh, Western General Hospital, Crewe Road, Edinburgh, UK

Supplemental information

- Supplementary Figure 1 – Page 1
- Supplementary Table 1 – Page 2
- Full methods – Page 3
- Primers – page 9
- References – Page 10

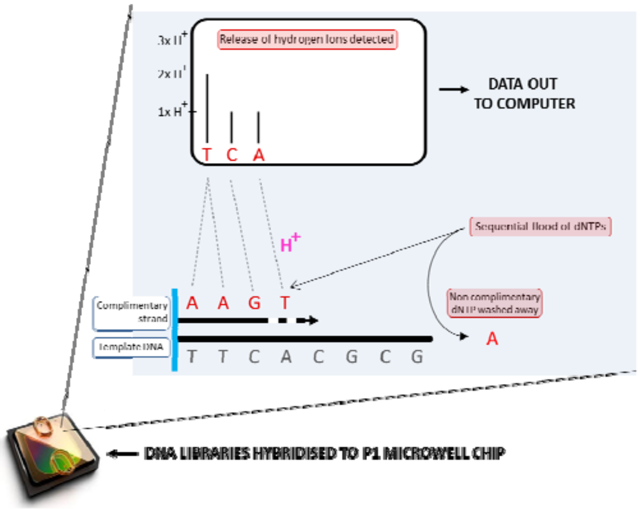

Supplementary Figure 1. An overview of the Ion Proton semiconductor sequencing technology. Single stranded DNA fragments with specific sequencing adapters are hybridised to a microwell late ("P1 chip") prior to sequencing to create a "template strand". The reaction chamber is then sequentially flooded with a single deoxyribonucleotide triphosphate (dNTP) in the presence of a DNA polymerase. If this dNTP is complimentary to the leading base on the template DNA strand it is incorporated on the complimentary strand and a single hydrogen ion is released which is read by an ion-sensitive field-effect transistor (ISFET) sensor and the reaction continues. If several dNTP molecules are incorporated in a single cycle (i.e if there is a series of particular bases on the template stand) this will lead to the release of multiple hydrogen ions which is read as a proportionally higher electronic signal by the ISFET.

Dataset	Sequencer	mark	raw read count	% mapped	Total read count	Fold coverage ǂ
MEF hmeDIP: *Reik et al 2011*[1]	Illumina genome analyzer IIx	5hmC	32,058,473	70.5%	22,594,128	2.23
MEF hmeDIP	Ion Proton SC seq	5hmC	25,688,226	85.0%	21,834,992	1.17
J1 ESC meDIP: *Reik et al 2011*[1]	Illumina genome analyzer IIx	5mC	21,171,139	42.8%	9,080,120	1.10
J1 ESC meDIP	Ion Proton SC seq	5mC	33,026,367	93%	30,714,521	1.64
**Liver datasets**						
hmeDIP liver A	Ion Proton SC seq	5hmC	43,018,696	90%	38,716,826	1.94
hmeDIP liver B	Ion Proton SC seq	5hmC	39,294,724	91%	35,758,199	1.78
hmeDIP MEF	Ion Proton SC seq	5hmC	25,688,226	85%	21,834,992	1.09
hmeDIP IPSC	Ion Proton SC seq	5hmC	33,945,549	90%	30,550,994	1.63
meDIP J1 ESC	Ion Proton SC seq	5mC	33,026,367	93%	30,714,521	1.54
Input liver A	Ion Proton SC seq	INPUT	37,328,357	91%	33,968,805	1.69
Input liver B	Ion Proton SC seq	INPUT	33,065,031	89%	29,427,878	1.47
Input MEF	Ion Proton SC seq	5hmC	38,439,367	85%	32,673,462	1.4
Input IPSC	Ion Proton SC seq	INPUT	44,364,403	90%	39,927,963	2.13
Input J1 ESC	Ion Proton SC seq	INPUT	36,249,604	93%	33,712,132	1.69

Supplementary table 1. Read count and fold coverage data for Ion Proton SC seq runs and for comparable Illumina sequenced mouse embryonic stem cells (J1 ESC), mouse embryonic fibroblast (MEF) , mouse induced pluripotent stem cells (IPSC). Values for the Hiseq data taken from [1]. Fold coverage values for Hiseq datasets (ǂ) are skewed due to the fact paired end sequencing was employed

Full methods

i. Hydroxymethyl and Methyl DNA immunoprecipitation (HmeDIP & MeDIP)

Typically 5µg of starting material is used, however lower amounts ~500ng can also be employed. Depending on the amount of DNA used the sonication conditions must be optimised. These will also differ depending on the device used to fragment the DNA. In our hands we have used the Diagenode Bioruptor to sonicate 20 µg of DNA in 400 µl TE. Genomic DNA (gDNA) is sheared to produced fragments ranging from 150bp-500bp with the majority of fragments 250-300bp. This is confirmed by electrophoresing 500ng out on a 1.5% agarose gel and post-staining with ethidium bromide.

5µg of fragmented gDNA is then taken and denatured at 100°C for 10 minutes (to subsequently allow the antibody to bind). At this stage 10% of the sample is then removed, cleaned up using DNA Clean & Concentrator™ kit (Zymo Research) and stored to be sequenced as the input. The DNA is now ready to be used for the analysis of either 5mC, or 5hmC profiles. For 5hmC analysis, the remaining sample is enriched for 5hmC through antibody enrichment following protocols outlined by [1]. In short, this relies on the binding of a specific 5hmC antibody (Active motif rabbit polyclonal against hydroxymethylation cat#39769) prior to enrichment through magnetic IgG beads (Dynabeads protein G (Invitrogen #100-03D). Following a series of buffer washes the DNA is then released through proteinase K digestion of the antibody and the DNA is subsequently cleaned up using Qiagen QIAquick PCR purification kits (Qiagen) and eluting in 20ul purified water.

ii. Amplification

Following enrichment, 10µl of the purified DNA is taken for whole genome amplification (WGA) using an enhanced amplification kit optimised for next generational sequencing (Sigma-Aldrich SeqPlex DNA Amplification Kit). The remaining 10µl is stored for later qPCR validation. WGA is carried out as per manufacturer's instructions with the exception of the number of cycles applied

during amplification. As we wish to limit overall amplification but to obtain sufficient double stranded DNA from the single stranded fragments enriched during hmeDIP, we limit the amplification to only 10 cycles. Primer removal is then carried out as per manufacturer's instructions and DNA cleaned up once more using the DNA Clean & Concentrator™ kit (Zymo Research) .

iii. Genome wide sequencing

a) Semiconductor sequencing

Amplified Input and 5hmc enriched DNA samples were analysed on the Bioanalyzer instrument with an Agilent High Sensitivity DNA Kit, to assess the fragment size distribution and to determine the volume of sample required for the Library preparation.

Individual libraries were generated from 100ng each of 5hmc enriched and input DNA for each sample, using the Ion XpressPlus Fragment Library Kit (life Technologies™). The DNA was end repaired, purified and then ligated to Ion-compatible barcoded adapters (Ion Xpress™ Barcode Adapters 1–96: Life Technologies™), followed by nick-repair to complete the linkage between adapters and DNA inserts. The adapter-ligated library was then amplified (10 cycles) and finally size-selected using two rounds of AMPure XP bead capture to size-select fragments approximately 100–250 bp in length.

An aliquot of each library was analysed on the Bioanalyzer instrument with an Agilent High Sensitivity DNA Kit, to assess the size distribution and determine the molar library concentration.

An equimolar pool of barcoded libraries was prepared at 100pM; each pool contained an hmc-DIP enriched and corresponding input sample. 8pM of the pooled library was added into an emulsion PCR based template reaction; in this reaction the fragments generated during the library prep were

attached to Ion sphere particles (ISPs) and clonally amplified. This process was carried out using the Ion One Touch 2 system and the Ion P1 Template OT2 200 Kit.

Quality control was performed on the Qubit 2.0 Fluorometer using the Ion sphere Quality Control assay. The optimal amount of library corresponds to the library dilution that gives percent template ISPs between 10–30%

The template positive ISPs were then enriched and sequenced on the Ion Proton using the Ion P1 Sequencing 200 Kit v3 and an Ion P1 chip.

b) Illumina Hiseq sequencing

Amplified Input and 5hmc enriched DNA samples were analysed on the Bioanalyzer instrument with an Agilent High Sensitivity DNA Kit, to assess the fragment size distribution and to determine the volume of sample required for the Library preparation. Library preparation and sequencing on 1/2 lane of an Illumina Hiseq 2000 (100bp sequencing) was carried out at the WASP sequencing facility, Albert Einstein College of Medicine Of Yeshiva University, New York.

iv. Bioinformatic processing

Reads generated by semiconductor sequencing were mapped to the mouse reference genome (mm10) using the Ion Torrent Suite software version 4.0.2, which utilizes the integrated TMAP aligner to produce a binary alignment map (BAM) file. BAM files were then converted first to BED files ("BAM to BED") and then into wiggle files ("In2Wig") using on our local GALAXY server. Following this we removed any small reads from the dataset as these may be incorrectly annotated due to the lower mapping accuracy scores (filter all reads <50bp in length). In order to directly

compare multiple datasets and to smooth out spurious reads we then carry out a sliding window analysis across the entire genome, calculating the average DNA modification scores in a non-overlapping set window size (Fig 4d). For the majority of epigenetic studies windows of 200bp are sufficient, however for higher density plots windows of 100bp can be applied, but will lead to a significant increase in the final size of the dataset. The end result is a series of genomic coordinates, each 200bp long containing levels of a given DNA modification across the genome. Due to the identical genomic coordinates they are therefore able to be directly interrogated by both Intra-chip (compare IP to input) and inter-chip (compare two IPs on separate chips) analyses.

Normalisation of the datasets can then be carried out. In our study we carried out read length normalisation to account for sequencing reactions which differed in efficiency, although other methods (such as quantile normalisation approaches), may also be used. As the IP and input are both sequenced on the same chip, internal normalisation can then be applied to each dataset. Much like a microarray this allows the user to subtract the values sequenced in the input from the IP, thus removing the background sequencing/DIP noise which allows for more accurate intra-array studies. As such positive read scores represent a true enrichment over input reads.

Resulting wiggle files were then visualised using software such as the Integrative Genomics Viewer (IGV) found at http://www.broadinstitute.org/software/igv/download. Mm10 derived datasets were also lifted over to mm9 builds to compare directly to published datasets.

v. Bioinformatic analysis

Following preparation and normalisation of the datasets we set out to analyse the files with respect to other sets of published mouse liver 5hmc data - both high resolution quarter genome hmeDIP microarrays produced by ourselves [2] as well as affinity purified and Illumina Hi-seq sequenced

5hmC datasets produced by others [3]. These datasets were first processed so that they were in a similar 200bp window format as our hmeDIP-SC-seq data (see above). In studies whereby we compared the hmeDIP-SC-seq data to microarrays the datasets were restricted to the regions covered by the array. Scatter plots of 5hmC levels at 500,000 random 200bp windows were drawn using the plot & smooth scatter functions in R for biological replicate hmeDIP microarray or hmeDIP-SC-seq whereby R2 values were calculated (Fig 3a). A Pearson's correlation matrix with clustering was then carried out between the microarray datasets (n=2), affinity hmeDIP Illumina seq dataset (n=1), a hmeDIP Hiseq dataset (n=1), the hmeDIP-SC-seq datasets (n=2) and matched input SC-seq datasets (n=2).

Average patterns of 5hmC were plotted across a length normalised gene set for the affinity Illumina seq dataset and the hmeDIP-Hiseq dataset as well as the two hmeDIP-SC-seq datasets. In short gene coordinates were normalised to a percentage length (TSS =0%, TES=100%) and then regions extended to cover 25% upstream and downstream. Genes <2kb in length were excluded from the analysis as were regions which overlapped with a nearby gene. Processed 5hmC patterns (average levels of reads across 200bp windows) were then plotted using the "sliding window over length normalised features" on our local GALAXY server, essentially plotting average patterns across and around genic portions of the genome. Following this analysis we identify "peaks " of 5hmC in the three 5hmC datasets by selecting windows which are above the 95th percentile of 5hmC values (the top 5% of the values) in the dataset for at least 3 consecutive probes (3x200bp windows=600bp). We then map these peaks to one of 5 genomic compartments (promoter core: TSS+/-100bp, promoter proximal: TSS +1kb, promoter distal: TSS+1kb to +2kb, Intra-genic: windows found uniquely within genic regions, intra-genic: windows found uniquely out with the aforementioned 4 regions) to test for similarities in genomic distributions of 5hmC between the two techniques.

Comparisons of the 5hmC patterns generated by hmeDIP-SC-seq were carried out to published Nimblegen deluxe 2.1M promoter microarrays for a mouse liver in which the mouse had been exposed to the non-genotoxic carcinogen , phenobarbital (PB) for 91days (for more info see [5]) where data was taken from GEO GSE40540.

Glucosylation mediated restriction enzyme sensitive qPCR (gRES-qPCR)

The EpiMark kit (NEB) was used to quantify relative levels of 5hmC and 5mC at select loci in mouse brain and liver DNA. All data was scaled at each locus so that the total % of marks = 100 and as such only relative and not absolute levels of each mark to be calculated. For the full protocol see the manufacturer's instructions. Typically, 10μg of genomic DNA was taken and half treated with T4-phage β-glucosyltransferase (BGT) for 12-16 hours at 37°C. Both the BGT treated and untreated samples were then divided into three PCR tubes and digested with either MspI, HpaII or left uncut for a further 12-16 hours at 37°C. Samples were proteinase K treated for 10 minutes at 40°C prior to dilution to 100μl final volume in H20 and heating to 95°C for 5 minutes. qPCR was carried out on 5 μl (~0.8μg DNA) of each sample on a Roche LightCycler 480 PCR machine. Relative enrichments of the modifications were then calculated following formulae provided by NEB.

Animal treatment and sample preparation

All animal procedures and Phenobarbital treatment were according to Thomson et al [6].

Reprogramming of MEFS

Conversion to induced pluripotent stem cells was carried out on mouse embryonic fibroblasts by established procedures [7].

Primers

Primer name	Fw	Rev
Gapdh Promoter	CCACTCCCCTTCCCAGTTTC	CCTATAAATACGGACTGCAGC
Act B promoter	ATGTACAGGAATAGCCTCCG	CTTAAGTGCTCGATATCCAC
Tex19.1 promoter	GGGAGATATGTAAATGAGCTGG	CATCCTTACCTCCCTGACTGAG
Prom1 promoter	GTTGCTCGAGCTTCTCCACT	GAAGGAGCCCAGCTTAGAGG
Cyp2b10 genic	TAAGTCCCATCCCTCTGTTC	GACAGACCCCATCTCAAAAA

Primer name	Fw	Rev
Gapdh Promoter	CCACTCCCCTTCCCAGTTTC	CCTATAAATACGGACTGCAGC
Act B promoter	ATGTACAGGAATAGCCTCCG	CTTAAGTGCTCGATATCCAC
Tex19.1 promoter	GGGAGATATGTAAATGAGCTGG	CATCCTTACCTCCCTGACTGAG
Prom1 promoter	GTTGCTCGAGCTTCTCCACT	GAAGGAGCCCAGCTTAGAGG
Cyp2b10 genic	TAAGTCCCATCCCTCTGTTC	GACAGACCCCATCTCAAAAA

References

1 Ficz, G. et al. Dynamic regulation of 5-hydroxymethylcytosine in mouse ES cells and during differentiation. Nature 473, 398-402 (2011).

2 Nestor, C. E. & Meehan, R. R. Hydroxymethylated DNA immunoprecipitation (hmeDIP). Methods Mol Biol 1094, 259-267, doi:10.1007/978-1-62703-706-8_20 (2014).

3 Thomson, J. P. et al. Comparative analysis of affinity-based 5-hydroxymethylation enrichment techniques. Nucleic Acids Res 41, e206, doi:gkt1080 [pii](2013).

4 Neri, F. et al. Genome-wide analysis identifies a functional association of Tet1 and Polycomb repressive complex 2 in mouse embryonic stem cells. Genome Biol 14, R91, doi:10.1186/gb-2013-14-8-r91 (2013).

5 Thomson, J. P. et al. Dynamic changes in 5-hydroxymethylation signatures underpin early and late events in drug exposed liver. Nucleic Acids Res 41, 5639-5654,(2013).

6 Thomson, J. P. et al. Non-genotoxic carcinogen exposure induces defined changes in the 5-hydroxymethylome. Genome Biol 13, R93,(2012).

7 Yusa, K. Seamless genome editing in human pluripotent stem cells using custom endonuclease-based gene targeting and the piggyBac transposon. *Nat Protoc* **8**, 2061-78 (2013).
